# Central nervous system imaging in childhood Langerhans cell histiocytosis – a reference center analysis

**DOI:** 10.1515/raon-2015-0024

**Published:** 2015-08-21

**Authors:** Luciana Porto, Stefan Schöning, Elke Hattingen, Jan Sörensen, Alina Jurcoane, Thomas Lehrnbecher

**Affiliations:** 1 Neuroradiology Department, Johann Wolfgang Goethe University, Frankfurt/Main, Germany; 2 Pediatric Hematology and Oncology, Hospital for Children and Adolescents, Johann Wolfgang Goethe University, Frankfurt/Main, Germany

**Keywords:** Langerhans cell histiocytosis, child, central nervous system, magnetic resonance imaging

## Abstract

**Background:**

The aim of our study was (1) to describe central nervous system (CNS) manifestations in children with Langerhans cell histiocytosis (LCH) based on images sent to a reference center and meeting minimum requirements and (2) to assess the inter-rater agreement of CNS-MRI results, which represents the overall reproducibility of this investigation.

**Methods:**

We retrospectively reviewed brain MRI examinations in children with LCH, for which MRI minimum requirements were met. Abnormalities were rated by two experienced neuroradiologists, and the inter-rater agreement was assessed.

**Results:**

Out of a total of 94 imaging studies, only 31 MRIs met the minimum criteria, which included T2w, FLAIR, T1w images before/after contrast in at least two different section planes, and thin post contrast sagittal slices T1w through the *sella*. The most common changes were osseous abnormalities, followed by solid enlargement of the pineal gland, thickened enhancing stalk and signal changes of the dentate nucleus. Whereas inter-rater agreement in assessing most of the CNS lesions was relatively high (κ > 0.61), the application of minimum criteria often did not allow to evaluate the posterior pituitary.

**Conclusions:**

The diversity of radiological protocols from different institutions leads to difficulties in the diagnosis of CNS abnormalities in children with LCH. Although the inter-rater agreement between neuroradiologists was high, not all the LCH manifestations could be completely ruled out when using the minimum criteria. Brain MRIs should therefore follow LCH guideline protocols and include T1 pre-gadolinium sagittal images, and be centrally reviewed in order to improve the comparison of clinical trials.

## Introduction

Langerhans cell histiocytosis (LCH) is a rare disease of the monocyte-macrophage system, seen mainly in children, but which can occur in any age group. The clinical presentation of the disease may range from a self-healing bone-lesion to multi-system life-threatening disease. The choice of appropriate therapy is therefore a significant challenge, with treatment options varying from watch-and-wait to intensive chemotherapy.^1^,^2^ The pathogenesis of LCH remains unresolved, with data supporting both malignant transformation and immune dysregulation.^1^,^3^

Whereas skin and bone lesions are the most frequent manifestations of LCH, central nervous system (CNS) lesions are less common, and a wide variety of CNS findings have been described by magnetic resonance imaging (MRI).^1^,^3^,^4^ Unfortunately, many centers often do not follow a standardized protocol when evaluating these patients, or are unaware of potential complications such as degenerative CNS changes.^5^ It is thus important to evaluate the reliability of MRI in diagnosing CNS changes. The aim of our study was (1) to describe CNS manifestations of LCH in children based on images sent to a reference center and meeting minimum requirements and, more importantly, (2) to assess the inter-rater agreement of CNS-MRI results, which represents the overall reproducibility of this investigation in this patient population.

## Patients and methods

### Patients and MR imaging

As the national reference center for German childhood LCH, we regularly receive clinical data and imaging studies from children and adolescents with LCH. We included in our analysis all children with biopsy proven LCH from whom we had received MRI studies during the 2-year period between 2012 to 2014; notably, children could have had also MRI studies performed prior to 2012. Since the centers where the patient was treated often used different MR protocols, we included only patients with the following MR image sequences in the analysis: (1) Sagittal T1-weighted (w) images post contrast, permitting evaluation of the infundibulum (the majority of the images did not include sagittal T1-w pre contrast, with the result that this sequence could not be included as a minimum criterion); (2) T2-w images, fluid attenuated inversion recovery (FLAIR), and (3) T1-w images pre and post contrast in at least two different section planes. Enlargement of the pineal gland was defined as described previously by Sumida *et al.*^6^

Single and multisystem LCH as well as CNS risk lesions were defined according to the guidelines of the LCH study protocols.^4^ The study has been approved by the local Ethics committee.

### Statistical analysis

In addition to the neuroradiologist who references cerebral MRI studies of children with LCH in Germany (LP), MRIs were assessed by a second senior neuroradiologist. The inter-rater agreement was evaluated by the percent agreement (uncorrected) and the Cohen’s Kappa κ index (corrected for chance effects). Substantial or almost perfect agreement was defined for κ values between 0.61–1.0.^7^ For the analysis, the software R Statistics 2.15.1 (http://www.R-project.org/) in combination with functions from the packages “irr” and “caret” were used.^8^ The results of both the reference neuroradiologist (LP) and the inter-rater agreement are reported.

## Results

### Patients’ characteristics

Clinical data and imaging studies were available in a total of 94 children with LCH. Cerebral MRI was performed in these children for a variety of reasons such as the presence of neurological abnormalities (*e.g.*, diabetes insipidus (DI)), for further evaluation in patients with involvement of the skull/craniofacial bones, or according to the physician’s discretion (*e.g.* the involvement of the mandibula or the cervical vertebrae). Only brain imaging studies of 31 patients (22 of whom were boys) met the inclusion criteria of the study. Eleven children suffered from unifocal and 5 other from multifocal bone disease. Localized LCH of the skull was seen in 11 patients. Nine children were affected by multisystem LCH. Eleven children suffered from DI, and 2 other children had endocrine disorders other than DI. With the exception of 2 children presenting with absence epilepsy and muscular hypotonia, respectively, no neurological abnormalities were detected in any patient (Table 1).

The median age of the patients at the time of cranial MRI was 7 years (range 0.5–17 years), and the median time between the diagnosis of LCH and cranial MRI was 70 days (range 0–10 years). 11 patients had not received treatment for LCH at the time of MRI.

### MR imaging and correlation with clinical data

The most common MRI changes were osseous (55%), followed by solid enlargement of the pineal gland (45%), a thickened enhancing stalk (32%) and signal changes of the dentate nucleus (29%). Occasionally, hyperintensity in the hippocampus, parenchymal/meningeal enhancement and white matter hyperintensity were observed.

### Tumorous/granulomatous lesions

#### Hypothalamic-pituitary axis involvement, hypothalamus

Enhancement and thickening of the pituitary stalk > 3 mm were seen in 10 patients (32%) (Table 1). Seven of the 12 available pre contrast MR sagittal images demonstrated loss of bright spot. Nine patients with abnormalities of the pituitary stalk suffered from DI, whereas 1 patient had no clinical manifestation of DI. Seven patients had DI and CNS risk lesions, of whom five patients had additional multisystem LCH.

#### Pineal abnormalities

Solid enlargement of the pineal gland with enhancement was observed in 14 patients (45%). Enlargement was defined when the height of the pineal gland was more than 3.5 mm in patients younger than two years and as more than 4.5 mm in patients older than 2 years, respectively.^6^ In 6 patients, abnormalities were seen in both the pituitary stalk and pinealis, whereas in 12 patients, only one of the structures was affected (pituitary stalk [4], pineal enlarged enhancement [8]).

#### Extra-axial space involvement

**FIGURE 1.**
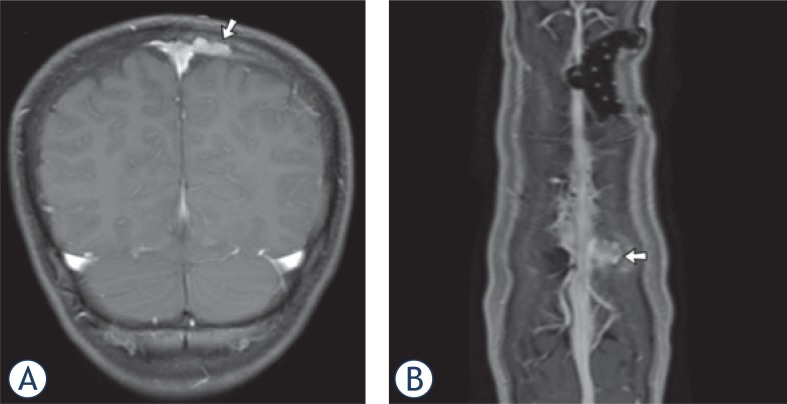
T1-w MR-images in an 11-year-old boy with LCH (patient ID #26). **(A)** Coronal enhanced T1-MR image reveals an osseous enhancing mass (arrow) combined with epidural und subdural involvement along the left side of the superior sagittal sinus. **(B)** Enhanced T1-MPRage-Image with reconstruction shows the mass (arrow) closely related to the superior sagittal sinus. Note the infiltration of the dural venous plexus, which is located within the inner portion of the dura. The dural plexus enhances in particular parasagittally on the left, where it connects to the sagittal sinus. There was no thrombus within the sagittal sinus.

4 patients (13%) presented with dural enhancement. All these patients had osseous lesions combined with epidural and subdural involvement (Figure 1). None of the patients presented with isolated dura-base masses or hypointensity on T2w at the choroid plexus.

#### Enhancement due to intracerebral granulomatous lesions

**FIGURE 2.**
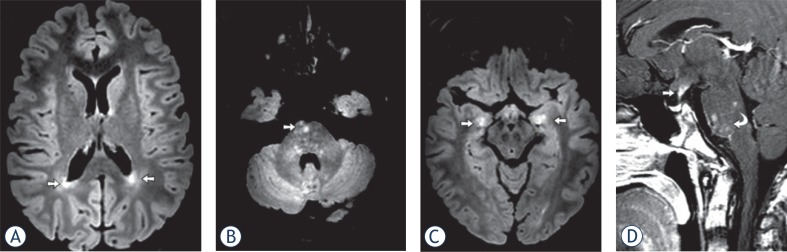
Cerebral MRI in a 15-year-old-boy with LCH (patient ID #4). **(A–C)** FLAIR images show high-signal lesions (arrows) in the deep white matter, in pons and hippocampus. After contrast note the parenchymal (partial nodular pattern, curved arrow) and perivascular enhancement. The classical finding, enhancement and thickening of the pituitary stalk, was also present (straight arrow).

**FIGURE 3.**
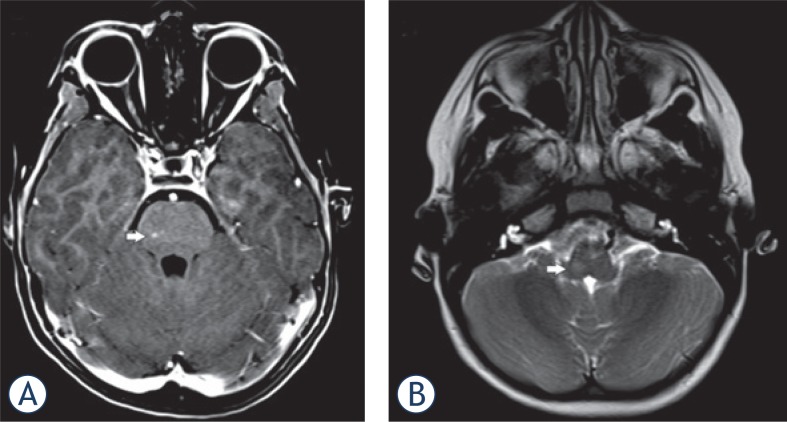
Cerebral MRI in a 8-year-old-boy (patient ID #8). **(A)** T1-w image after contrast shows enhancing lesions (arrow) in the pons. **(B)** Additional lesions were seen in medulla oblongata (arrow).

5 patients (16%) presented with parenchymal enhancement. Pontine enhancement was seen in 2 cases (Figures 2 and 3), and supratentorial enhancement in 4 patients. One patient presented with simultaneous infra- and supratentorial enhancement.

#### Lytic lesions of skull

Osseous lesions in the skull, skull base and craniofacial bones were seen in 17 patients (55%); of those, 4 had dural infiltration, 4 had extension of the disease within the intraconal space with enhancement, and 3 had bone destruction with involvement of the mastoid.

### Non-tumorous, non-granulomatous lesions

#### Dentate nucleus

The evaluation of the dentate nucleus as a classical location of deep grey matter affected by LCH was limited by artefacts resulting in a relatively low inter-rater agreement (κ < 0.61). None of the 9 children with dentate nucleus abnormalities showed neurological symptoms such as tremor, dysarthria, or ataxia. Whereas in 6 of the children, LCH had been diagnosed more than 1 year prior to the abnormal MRI finding; the affection of the dentate nucleus was seen in 1 untreated patient at the time of diagnosis of LCH.

#### White matter changes

A hyperintense signal on T2w and FLAIR images in the supratentorial white matter were observed in 2 patients (Figure 2) and were likely in 6 patients (representing a total of 25% of patients). The lesions were symmetrical and showed a vascular pattern. These white matter changes were associated with pons involvement in 2 patients, of which one showed enhancement (Figure 2).

#### Basal ganglia and hypothalamus

Changes in basal ganglia or in the hypothalamus were not observed in any patient.

### Atrophy

No signs of cerebellar, midbrain, or supratentorial atrophy were noted for any patient

### Inter-rater agreement

The diversity of CNS lesions may lead to difficulties in diagnosis, which may have a significant impact on treatment and outcome. The inter-rater agreement in this study was 69–100%. Substantial inter-rater agreement (κ > 0.61) was found for the following variables: enlarged pituitary stalk/mass, bone changes, pineal enlarged enhancement, white matter and hippocampal hyperintensity on T2w, as well as for parenchymal and meningeal enhancement. Partial volume effects and artefacts limited the MRI evaluation and the inter-rater reliability in the area of the dentate nucleus (κ = 0.31). Other variables, such as the hyper-intense signal on T2w and FLAIR in the lentiform nucleus, hypothalamus or cerebellum had low occurrence rates and are therefore unreliable agreement indices.

## Discussion

Since LCH is a rare disease, research on radiological CNS abnormalities is limited. In addition, the variable quality of diagnostics makes comparing treatment and outcome difficult. Our aim was to describe CNS manifestations in children with LCH based on images sent to a reference center and meeting minimum requirements. The second and more important goal was to evaluate the reliability of the MRI findings based on the inter-rater agreement of two senior neuroradiologists.

Only a total of 33% of the cerebral MRIs sent for reference met the minimum requirements of the study. Unfortunately, we had to omit pre contrast sagittal T1-w as a minimum criterion, since most imaging studies did not include this sequence. The lack of thin T1 sagittal pre contrast images can be explained by the fact that the German public health system only pays for a maximum of 4 MR sequences.

### 

#### Hypothalamic-pituitary axis

**FIGURE 4.**
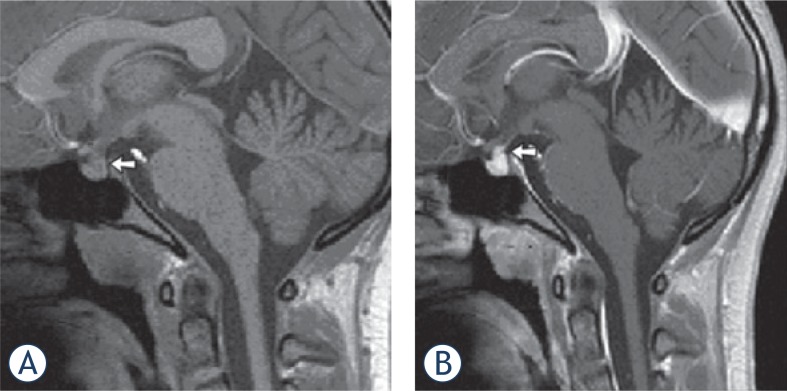
Sagittal T1-w MR image in a 14-year-old girl (patient ID #13). **(A)** T1-w image without contrast shows the typical lack of high signal intensity of the posterior pituitary (arrow). **(B)** After contrast note the enhancement and thickening of the pituitary stalk (arrow).

Diabetes insipidus, as the most common endocrinopathy in LCH, is caused by inadequate anti-diuretic hormone (ADH) secretion. Corroborating our data, DI occurs in approximately 25% of all patients with LCH or in approximately 50% of patients with multisystem disease, mainly in those with skull and orbital involvement.^9^–^12^ The typical MRI finding in DI is the lack of high signal intensity of the posterior pituitary on T1w images before contrast (“loss of bright spot”), which correlates with the loss of ADH-containing granules, and is often associated with enhancement and thickening of the pituitary stalk >3 mm (Figure 4).^13^ In our series, loss of bright spot was seen in 7 patients, but it is important to note that this result may be biased as usually only children with DI or anterior pituitary hormone deficiency have a typical targeted MRI examination of the pituitary region which includes T1w without gadolinium. Enhancement and thickening of the pituitary stalk were seen in 10 of 31 (32%) of our patients with high inter-rater agreement. Abnormalities of the pituitary stalk were present in 9 of the patients with DI, but notably, also in one patient without DI. Interestingly, over the three year follow-up period, this patient, who received prolonged chemotherapy for primary and relapsed LCH, did not develop DI. Future research is needed to address which children with LCH and associated abnormalities of the pituitary stalk will develop DI at a later time-point,^14^ and, more importantly, whether treatment in these children could prevent this specific complication.

#### Pineal abnormalities

Enhancement of the solid enlarged pineal gland was seen with a high inter-rater agreement in 14 patients (45%), which is considerably higher than reported previously (3% and 15%).^15^,^16^ This difference could be due to several reasons: Firstly, different MRI protocols make it difficult to evaluate pineal enhancement, and the lack of high-resolution thin-section imaging may result in false-negative findings. Secondly, the normal pineal gland tissue enhances with gadolinium on MRI because of the lack of a blood-brain barrier. This means that contrast enhancement within the pineal gland, especially in teenagers, does not necessarily mean abnormality, *e.g.* infiltration. There is a greater prevalence of ring-like pineal glands in children than in adults, and it was postulated that these glands may form pineal cysts in the future, which would account for the higher percentage enhancing mass in children and teenagers, and of cysts in adulthood and the increased incidence on autopsy reports.^17^ It is also important to note that the results of the pineal gland should be interpreted carefully, since there is a large variation of the size of the gland in all age groups.^6^

In contrast to a previous study, we did not find a correlation between abnormalities in the pituitary stalk and the pineal gland.^15^ Whereas in 6 patients of our series, both structures showed an abnormality, 12 patients presented with an irregularity in one structure only. It was speculated whether coexisting changes, which have also been observed in other diseases, might be caused by the functional interactions of both structures.^18^ However, as noted before, the intrinsic enhancement of the pineal gland makes the evaluation of this structure difficult.

#### Lytic lesions of skull

Craniofacial involvement is the most common presentation of CNS-LCH. In line with other reports, the frequency of these abnormalities in the present study was 55%.^19^,^20^

#### Intracranial non-tumorous lesions including neurodegenerative changes

Intra-axial neurodegenerative parenchymal changes are among the most frequent patterns of CNSLCH.^16^ Neurodegenerative grey-matter changes mainly involve the dentate nucleus and basal ganglia, with a bilaterally symmetrical, hyperintense signal in T1w and T2w as key radiological features.^4^ Alterations in the signal-intensity may reflect the loss of neurons, demyelination, gliosis, and inflammation.^4^,^21^ It was speculated that chronic or recurrent granulomatous lesions in the craniofacial bones result in an intracranial process which includes chemokine-mediated tissue damage or an autoimmune response to brain tissue induced by antigen-presentation through Langerhans cells.^4^ If this is the case, both the frequency and severity of neurodegenerative lesions might increase with older age of patients with LCH. This hypothesis could explain the lower frequency of grey-matter changes in the dentate nucleus in our study (29%) compared to a previous analysis (40%).^19^ The oldest patient in the present study was 17 years old, whereas Prayer et al included patients up to 47 years of age.^19^ In addition, the short follow-up is a limiting factor of our study. On the other hand, it is important to note that the inter-rater agreement assessing the dentate nucleus was moderate due to potential volume effects and artefacts. Interestingly, one 14-year-old patient demonstrated radiological signs of neurodegeneration without having received treatment for LCH. In contrast to a previous study, we did not see a correlation between radiological signs of neurodegeneration and pituitary involvement of LCH.^22^ We are currently designing a study which closely monitors children with LCH and radiological signs of neurodegeneration over a long period of time in order to identify risk factors for patients who will ultimately develop clinical symptoms of neurodegeneration.

#### Inter-rater variability

To date, no study has evaluated the inter-rater variability of CNS abnormalities of children with LCH. This, however, is important, since a valid evaluation is the prerequisite for comparing clinical trials. Although our study demonstrated a high inter-rater agreement for most of the MRI-findings in which minimal criteria were met, the data suggest that cerebral imaging should be centrally referenced as is the case for many pediatric tumors. The inter-rater agreement may even be significantly lower when MRI is performed outside a tertiary referral hospital or by a general radiologist. In addition, it would be interesting to evaluate the inter-rater variability between different reference centers, but this is beyond the scope of this study.

#### Limitations of this study

As in other studies in children with LCH, cerebral MRI was not performed in all patients, but only in those who had already developed or were at high risk for CNS complications, or according to the treating physician’s discretion. There was therefore an inherent selection bias as not all LCH patients had an MRI.

Although we included only patients with a minimum number of MR-image sequences, the study included imaging from various institutions with heterogeneous protocols making it difficult to assess all the CNS features of LCH. In addition, T1 pre-gadolinium imaging was not performed in most patients and it was therefore often not possible to assess the posterior pituitary bright spot.

## Conclusions

The majority of the CNS-images sent for reference did not follow previous guidelines^5^, and only one-third of the MRI could be included in this analysis. In order to improve the comparison of clinical trials in the future, all cerebral MRI should meet standardized protocols, which include the assessment of the posterior pituitary bright spot, and should be centrally reviewed. The following protocol is recommended: axial T2w, Fluid Attenuated Inversion Recovery (FLAIR) and T1 w of the entire brain; axial and coronal T1W post contrast of the entire brain (at least one with fat saturation to evaluate lesions of skull). In addition, the hypothalamo-pituitary region should be evaluated with ≤ 3 mm slice thickness with and without contrast enhancement.

In conclusion, (1) CNS manifestations are frequent, but result in variable findings in children with LCH, (2) the assessment of the posterior pituitary was not possible in most of the referred images, and (3), although the inter-rater agreement between neuroradiologists was high, the MRI diagnosis based on referred images was not suitable to rule out all LCH CNS manifestations.

## Figures and Tables

**TABLE 1.** Patients’ characteristics and cerebral magnetic resonance imaging (MRI) finding in 31 children and adolescents with Langerhans cell histiocytosis (LCH)

				**Pattern of LCH^1^**			**Clinical Symptoms**				**Cerebral MRI Abnormalities**		

**ID**	**Sex**	**Age at Imaging (yrs)**	**Time Diagnosis - Imaging**	**Single-system**	**Multi-system**	**CNS-Risk**	**DI**	**Endocrine (other than DI)**	**Other**	**Therapy^2^**	**Dentate nucleus**	**Pituitary stalk**	**Pineal**
1	M	17	<10 d	y			y					y	Y
2	M	7	<10 d	y		y				y			
3	M	11	<10 d	y						y			
4	M	15	10 yrs		y		y			y	Y	y	Y
5	F	3	170 d		y					y			Y
6	M	1	<10 d	y									
7	M	3	<10 d	y									
8	M	8	7 yrs		y		y			y		y	Y
9	F	9	18 mths	y		y			Absence seizures	y			Y
10	M	11	8 mths	y				Somatomegaly		y			Y
11	M	15	3 mths	y						y	Y		
12	F	3	3 yrs		y	y	y			y	Y		Y
13	F	14	2 mths	y			y				Y	y	
14	M	3	2 mths		y	y	y		Muscular hypotonia	y		y	Y
15	F	1	10 d	y		y							Y
16	M	3	2 yrs		y					y	Y		
17	M	15	3 yrs	y									
18	M	5	<10 d	y									Y
19	M	1	< 10d	y		y				y	Y		
20	F	10	5 yrs	y			y			y	Y		
21	M	9	<10 d	y		y							
22	F	0,5	< 10d		y	y				y			
23	M	3	2 yrs		y					y	Y		Y
24	F	10	2 mths	y						y			Y
25	M	17	3 yrs		y		y			y		y	
26	M	11	<10 d	y								y	
27	M	6	22 d	y						y			
28	M	5	3 yrs	y			y	Hypopituitarism		y	Y	y	Y
29	M	14	<10 d	y		y				y			
30	M	11	<10 d	y			y					y	
31	F	6	3 mths	y			y					y	y

y represents “present”, blanks represent “not present”.

1 The pattern of LCH single- and multi-system LCH as well as central nervous system (CNS) risk lesions were defined according to the guidelines of the LCH study protocols [Ref. 5];

2 Therapy given prior to or at the time of cerebral imaging consisted of regimens according to LCH or to modified LCH protocols; d = day; DI = diabetes insipidus; f = female; m = male; mth = month; yr = year
